# Compound heterozygosity in sodium channel Nav1.7 in a family with hereditary erythermalgia

**DOI:** 10.1186/1744-8069-4-21

**Published:** 2008-06-02

**Authors:** Mark E Samuels, Rene HM te Morsche, Mary E Lynch, Joost PH Drenth

**Affiliations:** 1 Département de Médicine, Centre de Recherche du CHUM, Local M-5226, Hôpital Notre-Dame, 1560 rue Sherbrooke Est, Montréal QC H2L 4M1, Canada; 2 Department of Medicine, Division of Gastroenterology, University Medical Center St. Radboud, PO Box 9101, 6500 HB Nijmegen, The Netherlands; 3 Departments of Psychiatry, Anesthesia and Pharmacology, Victoria General 10 West Victoria, 1278 Tower Road, Dalhousie University, Halifax NS B3H 2Y9, Canada; 4 Current address : A-733, Centre de Recherche du CHU Ste-Justine 3175, Cote Ste-Catherine Montreal, QC H3T 1C5 Canada

## Abstract

Hereditary erythermalgia is a painful and debilitating genetic disorder associated with mutations in voltage-gated sodium channel Nav1.7. We have previously reported a Canadian family segregating erythermalgia consistently with a dominant genetic etiology. Molecular analysis of the proband from the family detected two different missense mutations in Nav1.7. In the present study we have performed a long-term follow-up clinical study of disease progression in three affected family members. A more extensive molecular study has also been completed, analyzing the segregation of the two missense variants in the family. The two variants (P610T, L858F) segregate independently with respect to clinical presentation. Detailed genotype/phenotype correlation suggests that one of the two variants (L858F) is causal for erythermalgia. The second variant (P610T) may modify the phenotype in the proband. This is the second reported study of potential compound heterozygosity for coding polymorphisms in Nav1.7, the first being in a patient with paroxysmal extreme pain disorder.

## Findings

A Canadian family segregating erythermalgia as an apparent dominant genetic trait was originally ascertained and reported in 1979[1]. At the time, the female proband was 14 years old. She was reported to have two affected brothers, however only the proband's condition was described. She had already suffered double leg amputations at age 13, with regular severe episodes of pain and redness in the extremities. A brief report of the family in 1987 noted that the proband had suffered a near-fatal case of hypothermia due to excessive use of a cooling apparatus to relieve painful symptoms[2]. One brother had suffered two leg amputations at age 20. The family was subsequently included in a cohort of families used to map a locus for erythermalgia to chromosome 2q31-32[3], and eventually molecular analysis of the proband identified two missense variants in voltage-gated sodium channel Nav1.7, amino acids P610T and L858F[4]. Segregation of these two variants in the remaining affected and unaffected family members was not feasible at that time.

**Figure 1:**
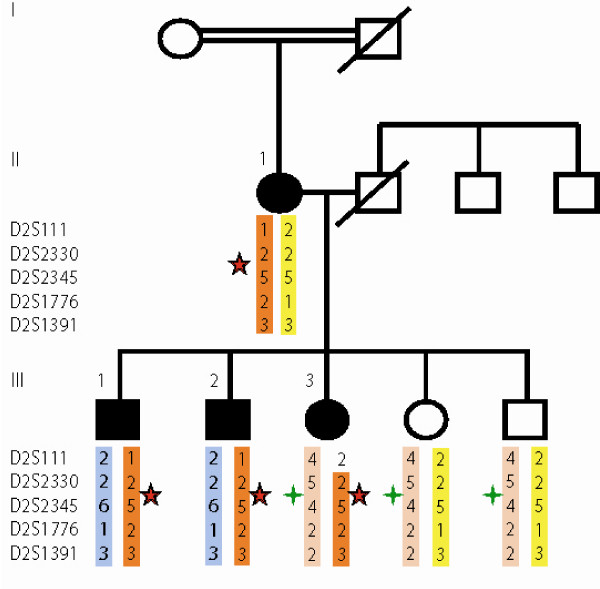
**Missense variants in Nav1.7 in erythermalgia family**. Canadian family with inherited primary erythermalgia. Colored bars represent derived haplotypes of the indicated microsatellite markers (haplos from deceased father are inferred). Segregation of two missense variants in Nav1.7, L858F (red star) and P610T (green star) are shown.

We have now revisited the affected family members in a long-term clinical follow-up (see Fig. 1).

Proband (III/3): The proband is a female now 43-years-old. She has continued to suffer extremely debilitating episodes of pain. On a scale of 1–10, with 10 the most extreme, she reports her pain as 10 in the absence of mitigating treatments. She has had no further amputations since the original ones at age 14. She reports burning, redness and pain in hands, arms and legs (above the amputations) as well as eartips, but not her nosetip. She does not suffer diarrhea. Symptoms are provoked by exercise or warm temperatures or use of bed sheets, and are more common during summertime. She is unable to wear shoes, and unable to work. Symptoms are incompletely alleviated by application of cold water or ice, air conditioning, elevation of the painful extremity, or by treatment with some medications.

Brother (III/1): The oldest brother is now age 49. He reports having symptoms from birth, with burning and reddened hands and ears. Both legs were partially amputated at age 20, he continues to experience symptoms in the legs in the vicinity of the stumps. Symptoms are provoked by heat or use of bed sheets and are alleviated by application of ice or use of a ventilator. He does not wear shoes and symptoms interfere with his ability to work or leave the home. He does not use pain medication.

Brother (III/2): The second brother is now age 48. He reports having symptoms from birth, with reddened and burning painful hands, feet, and ears. He reports warm and reddened but not painful nosetip. On a scale of 1–10 he reports pain at 8–9. He has suffered no amputations but has discolored and ulcerated skin in the affected regions. Symptoms are provoked by heat, exercise or use of bed sheets, and increase during summertime. He employs a ventilator but not ice or ice water for alleviation of symptoms. He does not wear shoes. He has worked sporadically and has experienced difficulties with adapting to inflexible work and school environments. Alcohol use exacerbates symptoms.

Overall, although it is difficult to assess given the subjective nature of painful sensation, the proband appears to experience significantly greater pain and discomfort than her two siblings. This is based on the patients reports according to a numeric rating scale, the need for stronger medication and the requirement for amputations at a young age. The development of a near fatal case of hypothermia resulting from attempts to cool the extremities in a search for relief is also suggestive of more severe pain.

Mother (II/1): The proband reports her mother was affected with symptoms from childhood, but the mother was unavailable for direct clinical examination.

In the original molecular study of this family the entire coding region of Nav1.7 was sequenced in DNA from the proband. Two missense mutations were reported, at nucleotides C1828A and C2572T, corresponding to amino acid changes P610T and L858F respectively. We have now performed mutation analysis for these two variants in all available family members, including the affected mother, three affected and two unaffected siblings (see Figure 1). The figure shows the segregation of the two amino acid variants, as well as the microsatellite haplotypes determined as part of the original fine mapping of the locus[3].

All three affected siblings and the affected mother carry the L858F variant. Somewhat surprisingly however, the second variant, P610T, is shared by the proband and the unaffected but not the affected siblings. Microsatellite haplotypes support the inference that the two variants are in *trans *in the proband. Presumably the P610T variant was transmitted from the unsampled father.

The L858F variant was independently reported in a Chinese patient with erythermalgia[5]. L858 is located within the DII/S4–S5 linker which is conserved among all mammalian sodium channels, indicating a critical role in controlling biophysical properties of these channels. Interestingly, a different mutation at the same residue, L858H, was found in a different Chinese family with erythermalgia[6]. Electrophysiological studies document that the L858F mutation affects the normal physiological function of the channel and leads to increased hyperexitability, as the mutant channel produces larger response to only small stimuli[7]. The mutant channel also recovers more quickly from inactivation. In addition, cooling decreases the current density, slows deactivation and increases ramp currents. This brings the function of the channel within the range of the wild type protein.

Our findings are intriguing with respect to genotype/phenotype correlations of missense variants in Nav1.7. To a first approximation, the disease in the family appears to be caused by inheritance of the L858F mutation. This is consistent with other genetic and functional studies of the gene. Of itself P610T has no obviously demonstrable phenotype in carriers in our pedigree. P610 is located within the cytosolic linker joining transmembrane segment S6 of domain I and transmembrane segment S1 of domain II and appears to be located within a conserved region of SCN9a. However residues 609–611, though partly conserved, are not invariant among vertebrate species (see Table 1). Moreover, we found P610T in 10 out of 210 control Caucasian chromosomes. This further suggests that P610T is not a highly penetrant pathogenic mutation. The question remains whether P610T is able to modulate e.g. exacerbate the phenotype of the proband carrying the main causal variant, or to subtly influence sensitivity to pain independently. This could arise through either of two possibilities. First, P610T may be a very weak gain of function (gof) allele of Nav1.7. Second, P610T may be a loss of function (lof) allele. Human patients carrying loss of function alleles of Nav1.7 suffer a rare genetic hypoalgesia disorder called congenital indifference to pain (CIP) [8-10]. However CIP is recessive, so that affected patients are either homozygous or compound heterozygous for two, normally nonsense, mutations in the gene. Heterozygous carriers of Nav1.7 lof alleles have normal pain thresholds, suggesting the absence of a strong dose-sensitivity for Nav1.7. In the presence of a presumptive hyperalgesic gof allele (such as L858F), the occurrence of an lof allele in *trans *could potentially exacerbate the phenotype, due to the reduction in wild type functional gene product. Our suggestion of functional consequences of P610T is necessarily preliminary. A direct test through *in vitro *electrophysiology is beyond the scope of the present study. Such a test, complemented by population based association tests of differential pain sensitivity, would be required to explore our hypotheses directly.

**Table 1:** The P610T *SCN9A *mutation detected in this study is in bold, and for comparison the corresponding amino acids from various species are included. Note that PPM residues 609–611 are highly but not totally conserved among mammalian species.

*Homo sapiens*	NISQASRSP**P**MLPVNGKMHSA
*Macaca mulatta*	NISQASRSP**P**ILPVNGKMHSA
*Equus caballus*	NISQASRSP**P**MLPVNGKMHSA
*Bos taurus*	NISQASRSP**P**VLPVNGKMHSA
*Canis familiaris*	NISQASRSP**P**VLPVNGKMHSA
*Rattus norvegicus*	NISQASRSP**P**VLPVNGKMHSA
*Mus musculus*	NISQASRSP**P**VLPVNGKMHSA
*Cavia porcellus*	NISQASRSP**P**MLPVNGKMHSA
*Dasypus novemcinctus*	NISQASRSP**P**MLPVNGKMHSA
*Monodelphis domestica*	NISQASRSPRMLPVNGKMHSA
*Ornithorhyncus anatinus*	NLSQASRSLRMLPVNGKMHST
*Gallus gallus*	NISQASRPLTLFPVNGKMHST

The genetics of Nav1.7 provides substantial complexities. Broadly, mutations in the gene lead to one of three different phenotypes[11,12]. As noted, lof alleles lead to recessive hypoalgesia. A class of aberrant, or gof mutations, confirmed in many cases by electrophysiological studies, cause erythermalgia[4-7,13-24]. A third class of alleles cause another hyperalgesia condition, known originally as familial rectal pain and now as paroxysmal extreme pain disorder (PEPD) [25-27]. PEPD alleles of Nav1.7 are likewise missense variants, suggesting that they also have aberrant function (gof). They cause spontaneous rectal, ocular or submandibular pain with reddening. The reasons for the different tissue distribution of the symptoms of erythermalgia and PEPD remain to be determined. The causal alleles for each condition are distributed across various regions of the gene (see Figure 1 of Drenth and Waxman[12]), although most mutant PEPD alleles are further C-terminal than most erythermalgia alleles. Expression and further electrophysiological studies have partly clarified this issue, with subtle differences in *in vitro *biophysical effects of PEPD versus erythermalgia mutant alleles. One likely case of compound heterozygosity for two missense Nav1.7 alleles was reported in a PEPD patient[25], making this the second report of a potential compound heterozygote for two mutant alleles in this gene.

A challenge with interpretation of the case literature on treatment of these diseases is the occurrence of non-genetic disorders with very similar presentation (i.e. phenocopies). Particularly in the case of erythermalgia, there is an extensive literature on other causes of the condition, although in most cases a potential genetic etiology was probably not explored or even discussed[28]. So far, genetic analyses of familial and sporadic cases have been mostly restricted to cases with known childhood onset, on the presumption that adult onset is more likely to be caused by other non-genetic factors. This remains an assumption however, and will require further molecular genetic study. There are clearly many additional questions to answer about the role of Nav1.7 in sporadic and provoked pain sensation.

## Competing interests

The authors declare that they have no competing interests.

## Authors' contributions

Dr. S coordinated the study and drafted the manuscript, Dr. D supervised molecular genetic experiments, RtM performed molecular genetic experiments, Dr. L performed clinical studies of the patients. All authors have read and approved the final manuscript.

## Consent

Approval for this project was obtained from the research ethics board of the Queen Elizabeth II hospital at Dalhousie University. Written informed consent was obtained from the patients for the work and for this publication.
